# Long noncoding RNA and messenger RNA abnormalities in pediatric sepsis: a preliminary study

**DOI:** 10.1186/s12920-020-0698-x

**Published:** 2020-03-10

**Authors:** Zhenjiang Bai, Yiping Li, Yanhong Li, Jian Pan, Jian Wang, Fang Fang

**Affiliations:** 1 Pediatric Intensive Care Unit, Children’s Hospital of Soochow University, Suzhou, China; 2 Institute of Pediatric Research, Children’s Hospital of Soochow University, Suzhou, China; 3 Department of Nephrology, Children’s Hospital of Soochow University, Suzhou, China

**Keywords:** Pediatric sepsis, Long non-coding RNA, Messenger RNA, Expression profile

## Abstract

**Background:**

Sepsis represents a complex disease with dysregulated inflammatory response and high mortality rate. Long noncoding RNAs (lncRNAs) have been reported to play regulatory roles in a variety of biological processes. However, studies evaluating the function of lncRNAs in pediatric sepsis are scarce, and current knowledge of the role of lncRNAs in pediatric sepsis is still limited. The present study explored the expression patterns of both lncRNAs and mRNAs between pediatric sepsis patients and healthy controls based on a comprehensive microarray analysis.

**Methods:**

LncRNA and mRNA microarray was used to detect the expression of lncRNAs and mRNAs in the septic and control groups. Aberrantly expressed mRNAs and lncRNAs identified were further interpreted by enrichment analysis, receiver operating characteristic (ROC) curve analysis, co-expression network analysis, and quantitative real-time PCR (qPCR).

**Results:**

A total of 1488 differetially expressed lncRNAs and 1460 differentially expressed mRNAs were identified. A co-expression network of the identified lncRNAs and mRNAs was constructed. In this network, lncRNA lnc-RP11-1220 K2.2.1–7 is correlated with mRNA CXCR1 and CLEC4D; lncRNA lnc-ANXA3–2 is correlated with mRNA CLEC4D; lncRNA lnc-TRAPPC5–1 is correlated with mRNA DYSF and HLX; lncRNA lnc-ZNF638–1 is correlated with mRNA DYSF and HLX. Significantly different expressions between pediatric sepsis patients and controls were validated by qPCR for the 4 lncRNAs and 4 co-expressed mRNAs, validating the microarray results.

**Conclusions:**

Our study contributes to a comprehensive understading of the involvment of lncRNAs and mRNAs in pediatric sepsis, which may guide subsequent experimental research. Furthermore, our study may also provide potential candidate lncRNAs and mRNAs for the diagnosis and treatment of pediatric sepsis.

## Background

Long noncoding RNAs (lncRNAs) have been reported to play regulatory roles in a variety of biological processes [1–4]. Recent studies have shown that abnormal expressions of lncRNAs are involved in many inflammation-related diseases [5–9]. Sepsis represents a complex disease with dysregulated inflammatory response and high mortality rate. It is the world’s leading killer of children [10]. However, studies evaluating the function of lncRNAs in pediatric sepsis are scarce, and current knowledge of the role of lncRNAs in pediatric sepsis is still limited.

In the current study, we investigated the expression patterns of both lncRNAs and mRNAs between pediatric sepsis patients and healthy controls based on a comprehensive microarray analysis. Aberrantly expressed mRNAs and lncRNAs identified were further interpreted by enrichment analysis, receiver operating characteristic (ROC) curve analysis, co-expression network analysis, and quantitative real-time PCR (qPCR). Our research contributes to a comprehensive understading of the involvment of lncRNAs and mRNAs in pediatric sepsis, which may guide subsequent experimental research. Furthermore, our study may also provide potential candidate lncRNAs and mRNAs for the diagnosis and treatment of pediatric sepsis.

## Methods

### Sample preparation

Ten pediatric sepsis patients and 12 children who were scheduled for minor elective surgery such as circumcision or inguinal hernia repair as the control group were included (Table 1, Additional file 1). All the heparinized blood samples were obtained from Children’s Hospital of Soochow University. The study procedure was approved by the ethics committee of Children’s Hospital of Soochow University. The written informed consent was obtained from each participating individual’s guardian. Mononuclear cells (MNCs) were isolated, and then stored at − 80 °C before RNA extraction. The comparison of clinical characteristics between study groups was carried out using Mann–Whitney U test for continuous variables and Fisher’s exact test for categorical variables.

**Table 1:** Clinical characteristics of the sepsis and control groups used in both microarray and qPCR validation

Characteristic	Sepsis	Control	*P*
Number	10	12	
Age, median months [range]	4.32 [1.17–96.60]	12.95 [5.80–20.13]	0.10^a^
Gender			0.57^b^
Male	8	11	
Female	2	1	
Infection site			
Lung (%)	2 (20.0)	–	–
Brain (%)	4 (40.0)	–	–
Others (%)	4 (40.0)	–	–
Septic shock (%)	3 (30.0)	–	–
ICU stay, median hours [range]	273.50 [23.00, 1009.00]	–	–
ICU mortality (%)	3 (30.0)	–	–

^a^ *P* value of the Mann–Whitney U test

^b^ *P* value of the Fisher’s exact test

### LncRNA and mRNA microarray

Total RNA was extracted using RNAiso (TaKaRa, Dalian, China) from the 10 septic children and 12 controls. Affymetrix Human oelncRNA Array (v1.0, containing 91,363 lncRNAs and 27,134 coding transcripts) was used to detect the expression of lncRNAs and mRNAs in the septic and control groups. Total RNAs purity and concentration were evaluated by NanoDrop 2000 spectrophotometer (Thermo Scientific). RNA integrity was detected by capillary electrophoresis. Aaccording to manufacturers’ instructions, sample preparation, microarray labeling and hybridization were performed. In brief, total RNAs from cells were reverse transcribed to double strand cDNAs and then synthesized, labeled, and hybridized onto the LncRNA and mRNA microarray.

### Data analysis methods

Raw data generated using the Affymetrix Human oelncRNA Array from 12 normal controls and 10 septic patients were stored in. CEL files, and then pre-processed (background correction, quantile normalization, log2 transformed) using the Robust Multichip Average (RMA) method of the R package “affy” [11]. The R package “limma” was used to identify differentially expressed transcripts [12, 13] according to the criteria: (a) absolute Log2 Fold Change (LFC) was more than 1; (b) for the Wilcoxon test false discovery rate (FDR)-adjusted *P*-value was less than 0.05.

Functional interpretation (both gene ontology (GO) analysis and Kyoto Encyclopedia of Genes and Genomes (KEGG) pathway analysis) of the differentially expressed genes identified was further carried out using DAVID 6.8 [14, 15]. In GO analysis, in order to identify significantly enriched GO terms, a *P* value threshold of 0.05 was used [16]. In pathway analysis, enrichment analysis was performed by the hypergeometric test with a *P* value threshold of 0.05 based on the KEGG database [17]. ROC curves were used to assess the classification performance of lncRNAs and single coding genes [18]. In addition, according to the expression levels, differentially expressed lncRNAs and mRNAs were also calculated using pearson correlation coefficient. The correlation threshold was set to > 0.98. Then, the eligible correlated lncRNA-mRNA pairs were choosed to construct a co-expression network by Cytoscape 3.4.0 software [19]. Moreover, conservation analysis was also performed using the R package “phastCons100way.UCSC.hg19” [20–22].

### Quantitative real-time PCR validation

The remaining portion of microarray samples was used for quantitative real-time PCR validation. The RNA was reverse-transcribed using oligo-dT and mouse mammary tumor virus reverse transcripatase. qPCR was carried out with SYBR Green master mix. Primers designed were listed in Additional file 2. Transcript expression was normalized to β-actin mRNA. Relative expression of transcript was calculated according to the 2^-ΔΔCt^ method. Mann–Whitney U test was carried out to determine the expression difference between septic children and control group. Statistical analyses were carried out using GraphPad Prism software (GraphPad Software Inc.). All *P* values are two-sided. *P* < 0.05 was considered as statistically significant.

## Results

### Differetially expressed lncRNAs and mRNAs identified

**Fig. 1:**
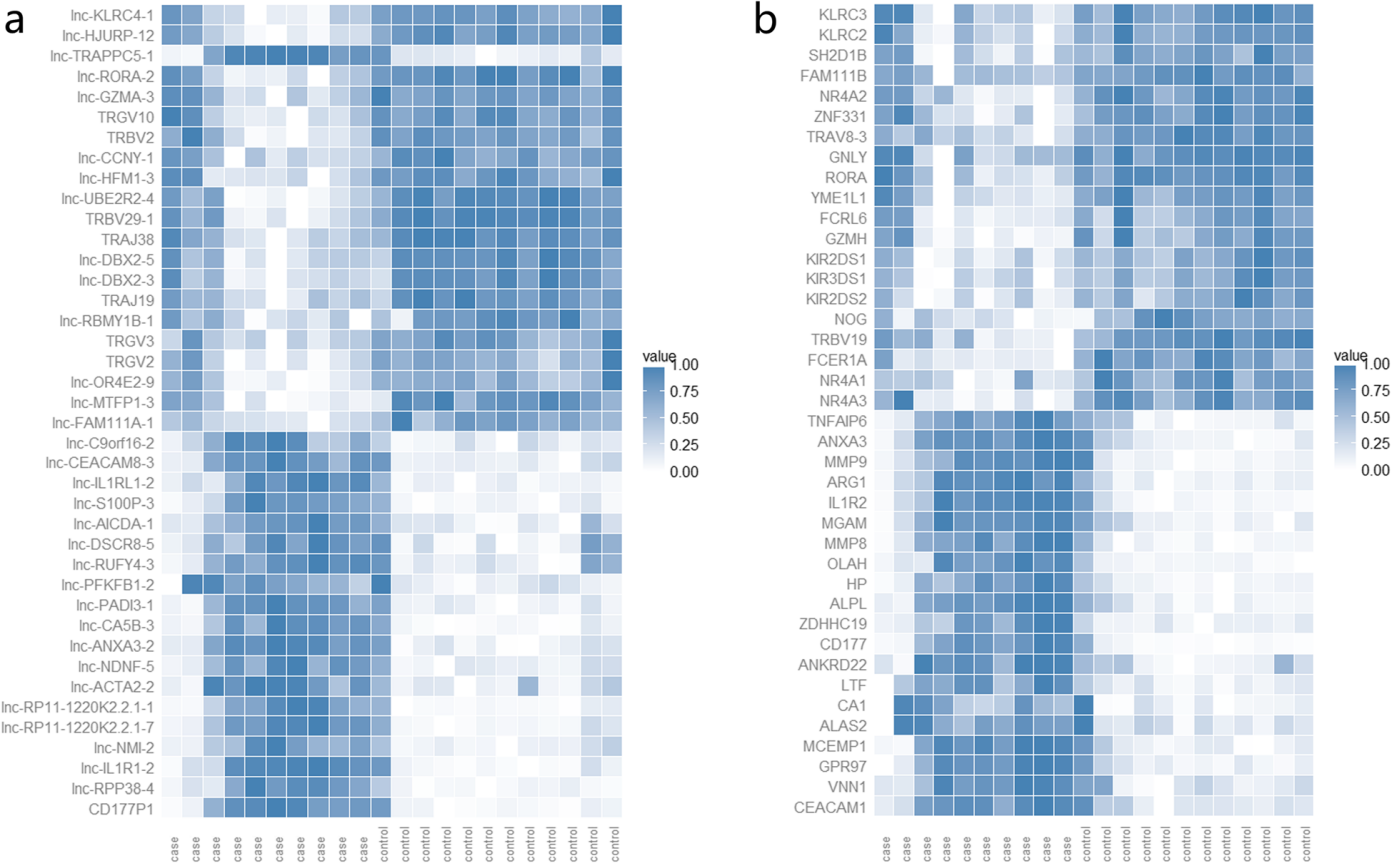
Heatmaps of expression data for the top 20 upregulated and top 20 downregulated **a.** lncRNAs and **b.** mRNAs

Ninety-one thousand three hundred sixty-three lncRNAs in total were detected, among which 1488 differetially expressed lncRNAs (996 upregulated; 492 downregulated, Additional file 3) were identified from 10 pediatric sepsis patients and 12 normal controls using the Affymetrix Human oelncRNA Array. The top 20 upregulated and top 20 downregulated lncRNAs are shown in Fig. 1a. lnc-RPP38–4 and lnc-HFM1–3 were the most upregulated and downregulated lncRNAs.

A total of 1460 differentially expressed mRNAs were also identified (1018 upregulated; 442 downregulated, Additional file 4) using the Affymetrix Human oelncRNA Array between pediatric sepsis group and control group. The top 20 upregulated and downregulated mRNAs are shown in Fig. 1b. MMP8 and FCER1A were the most upregulated and downregulated mRNAs.

### Functional annotation of differentially expressed lncRNAs and mRNAs

**Fig. 2:**
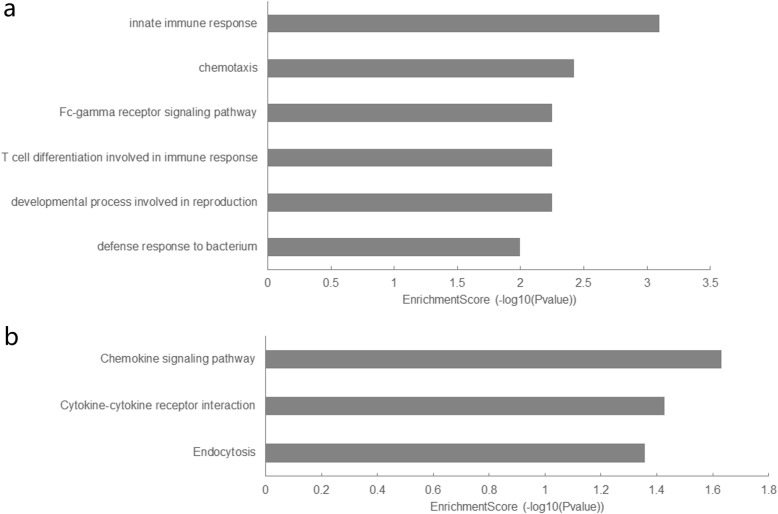
Summary of the **a.** Gene Ontology and **b.** pathway analysis results

The 1460 differentially expressed mRNAs underwent further functional investigation (GO analysis and pathway analysis). Figure 2 presented a summary of the GO and pathway analysis results. In the GO analysis, the top GO biological process term enriched was “innate immune response”. In the pathway analysis, the most significant pathway identified was chemokine signaling pathway, when we mapped the mRNAs to the KEGG database.

**Fig. 3:**
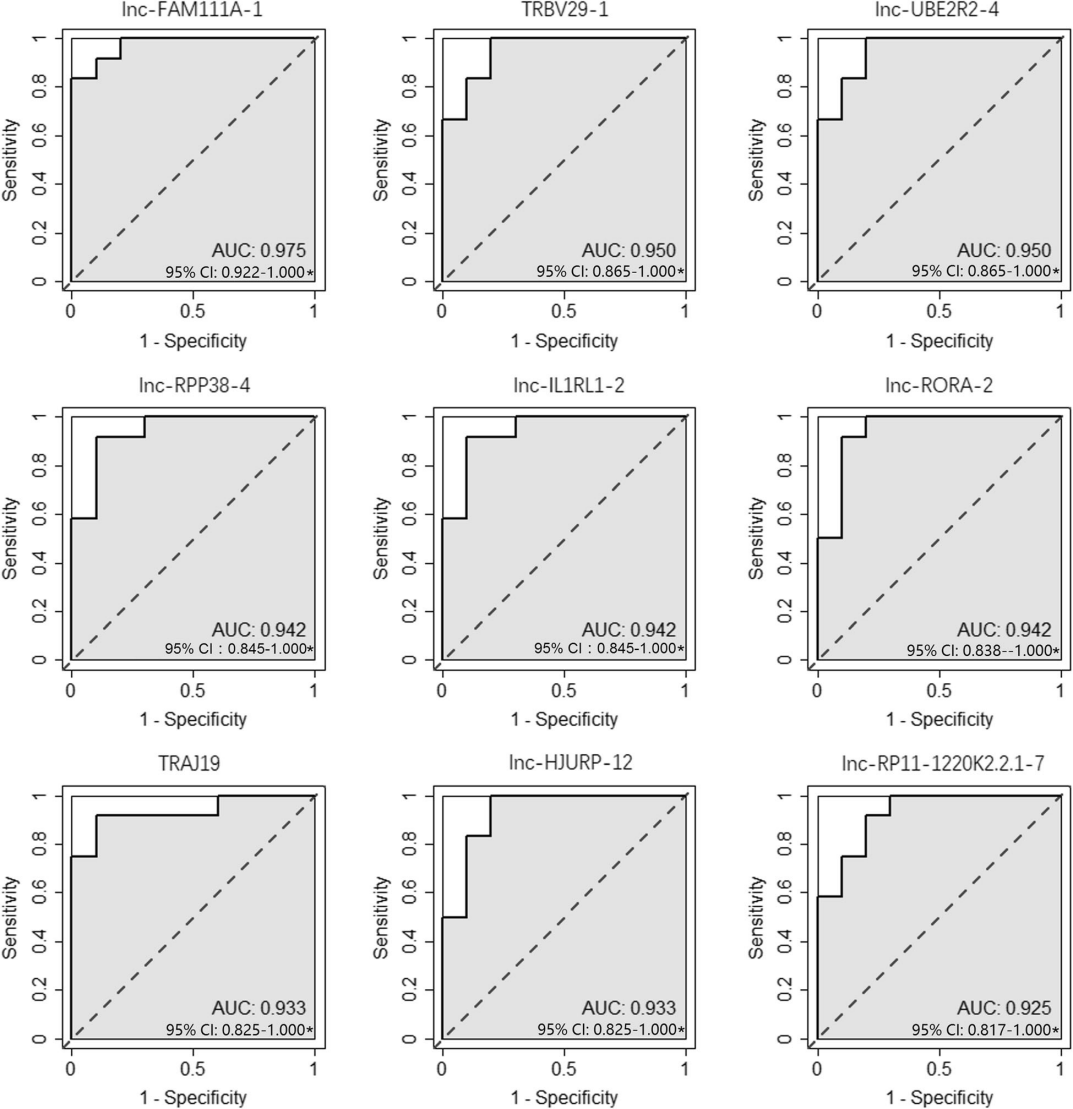
Top performing lncRNAs in diagnostic prediction of pediatric sepsis CI: confidence interval *: *P* < 0.01.

As for diagnostic prediction quality, ROC analysis results indicated that the top performing mRNAs were NLRC3, TMEM204, SPPL2A, UBASH3A, ARHGAP29, FKBP9, MTUS1, LCK, DAAM2 (Additional file 5), and the top performing lncRNAs were lnc-FAM111A-1, TRBV29–1, lnc-UBE2R2–4, lnc-RPP38–4, lnc-IL1RL1–2, lnc-RORA-2, TRAJ19, lnc-HJURP-12, lnc-RP11-1220 K2.2.1–7 (Fig. 3).

### LncRNA-mRNA co-expression network

**Fig. 4:**
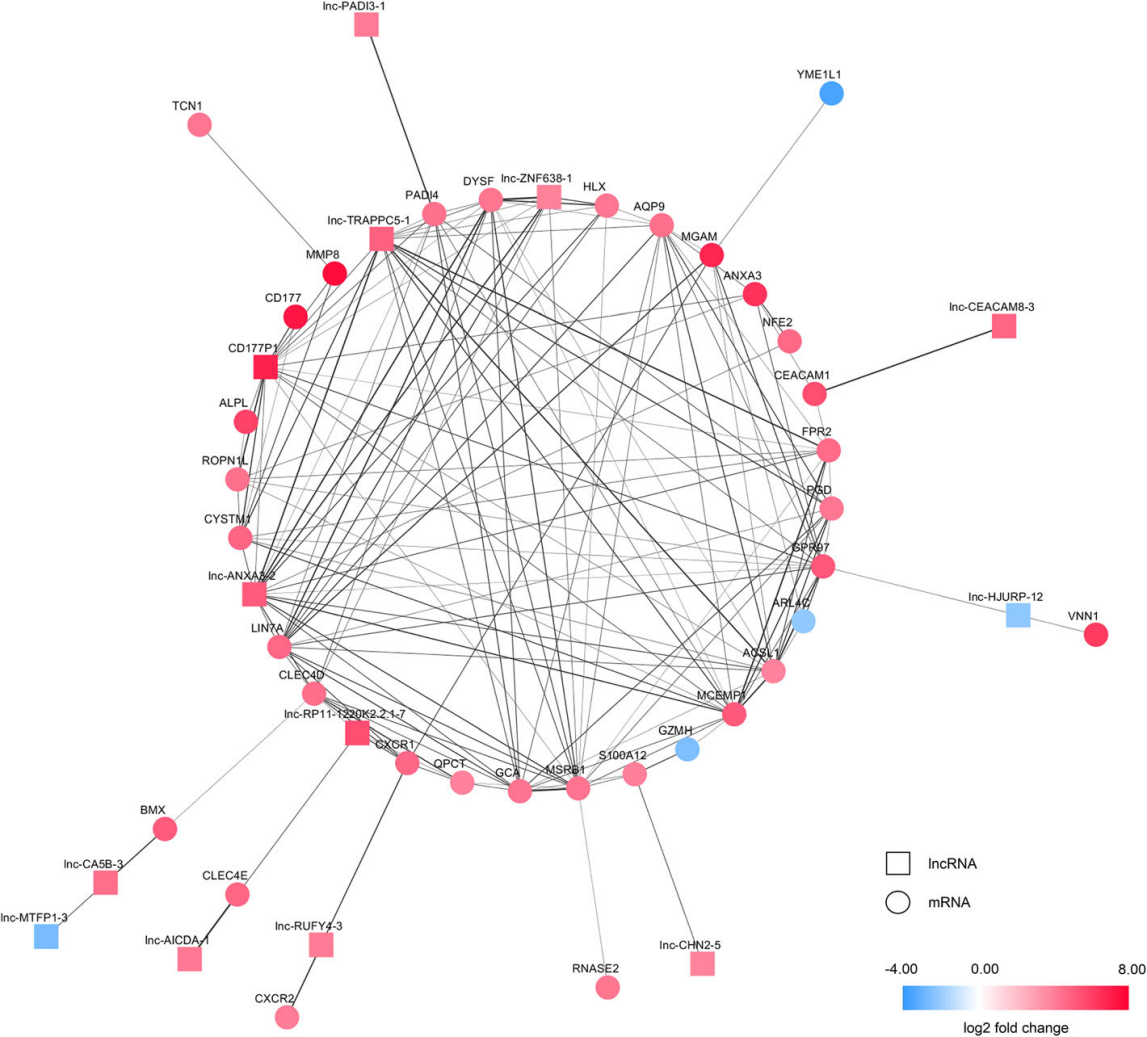
Co-expression network of the identified lncRNAs and mRNAs. Edge widths are proportional to the correlation coefficients

A co-expression network of lncRNAs and mRNAs was constructed using highly correlated transcript pairs (correlation coefficients > 0.98), which included 47 nodes and 177 edges (Fig. 4). As shown in Fig. 4, lncRNA lnc-RP11-1220 K2.2.1–7 is correlated with mRNA C-X-C motif chemokine receptor 1 (CXCR1) and C-type lectin domain family 4 member D (CLEC4D); lncRNA lnc-ANXA3–2 is correlated with mRNA CLEC4D; lncRNA lnc-TRAPPC5–1 is correlated with mRNA dysferlin (DYSF) and H2.0 like homeobox (HLX); lncRNA lnc-ZNF638–1 is correlated with mRNA DYSF and HLX. Conservation analysis was carried out and the conservation scores of the 4 lncRNAs (lncRNA lnc-RP11-1220 K2.2.1–7, lncRNA lnc-ANXA3–2, lncRNA lnc-TRAPPC5–1, lncRNA lnc-ZNF638–1) indicate weak conservations (Additional file 6).

Enrichment analysis of the co-expression network was also performed. The GO analysis results suggested that mRNAs in the co-expression network were enriched in innate immune response, chemotaxis, defense response to bacterium, interleukin-8-mediated signaling pathway, inflammatory response, T cell differentiation involved in immune response, Fc-gamma receptor signaling pathway, developmental process involved in reproduction, leukocyte migration, and dendritic cell chemotaxis. These results implicated that lncRNAs of the co-expression network correlated with mRNA expression and involved in the pathogenesis of sepsis.

### LncRNA & mRNA expression was validated by qPCR

**Fig. 5:**
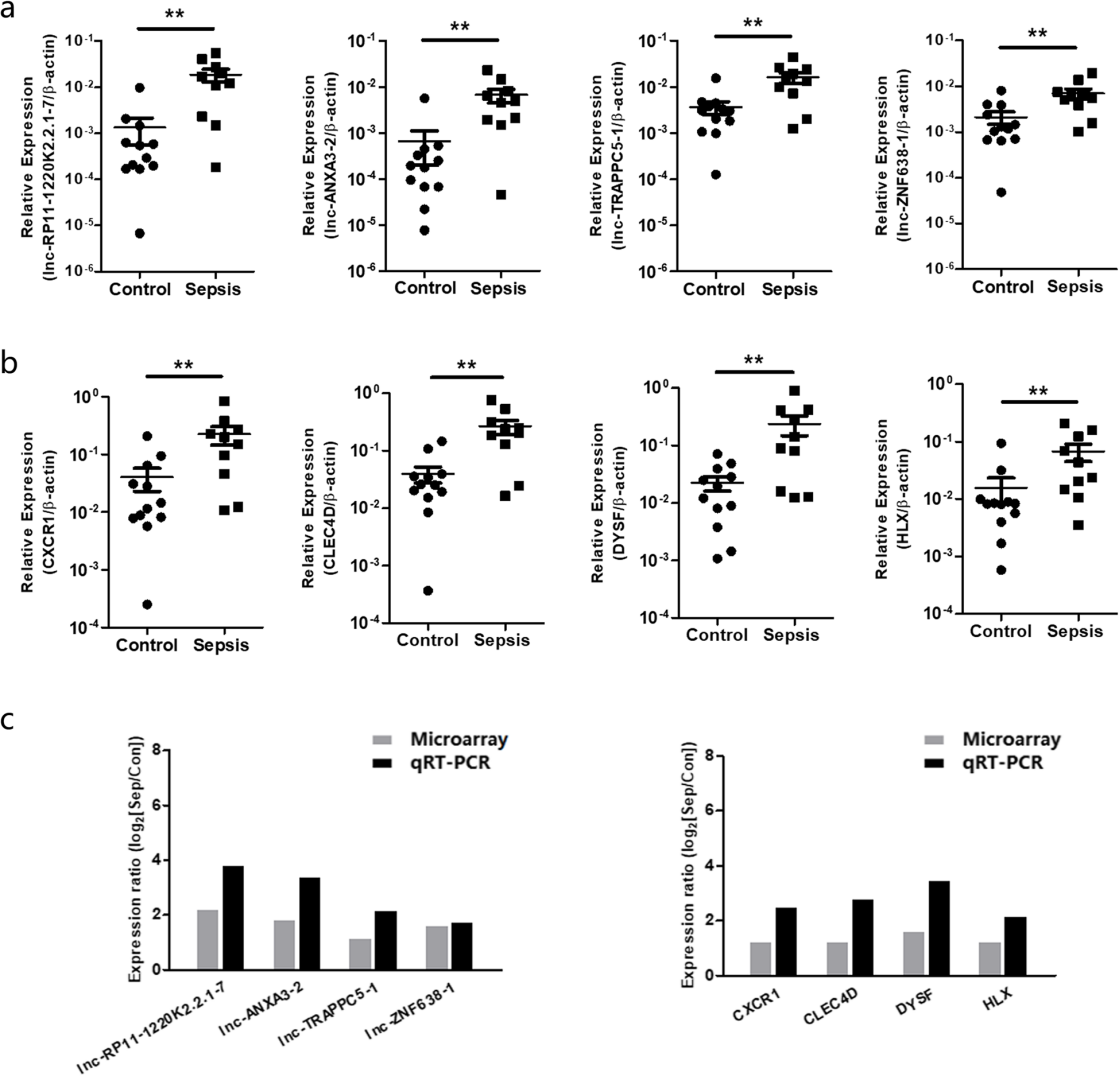
qPCR validation of 4 lncRNAs and 4 co-expressed mRNAs selected in the co-expression network. **a.** Relative expression comparison of 4 lncRNAs (lncRNA lnc-RP11-1220 K2.2.1–7, lncRNA lnc-ANXA3–2, lncRNA lnc-TRAPPC5–1, lncRNA lnc-ZNF638–1) between pediatric sepsis patients and controls (Mann-Whitney U test, ** *P* < 0.01). **b.** Relative expression comparison of 4 co-expressed mRNAs (CXCR1, CLEC4D, DYSF, HLX) between pediatric sepsis patients and controls (Mann-Whitney U test, ** *P* < 0.01). **c.** The qPCR results were similar to those acquired from microarray

To validate the expression changes of lncRNAs and mRNAs in pediatric sepsis detected by microarray, the 4 lncRNAs (lncRNA lnc-RP11-1220 K2.2.1–7, lncRNA lnc-ANXA3–2, lncRNA lnc-TRAPPC5–1, lncRNA lnc-ZNF638–1) and 4 co-expressed mRNAs (CXCR1, CLEC4D, DYSF, HLX) in the co-expression network (see Fig. 4) were further selected for qPCR validation using the remaining portion of microarray samples. As presented in Fig. 5, the expression levels of the 4 lncRNAs and 4 mRNAs in the sepsis group were significantly higher than those of the control group (*P* < 0.01 respectively, see Fig. 5a and Fig. 5b). The qPCR results were similar to those acquired from microarray (Fig. 5c), suggest that the 4 lncRNAs (lncRNA lnc-RP11-1220 K2.2.1–7, lncRNA lnc-ANXA3–2, lncRNA lnc-TRAPPC5–1, lncRNA lnc-ZNF638–1) are significantly highly expressed in septic children and could be novel biomarkers for pediatric sepsis.

## Discussion

Recent researches suggest that abnormal expressions of lncRNAs play roles in many inflammation-related diseases [5–9]. Comprehensive microarray analysis enables both lncRNA and mRNA screening in pediatric sepsis, which has the likelihood of discovering candidate lncRNAs and mRNAs for diagnosis and treatment.

In this study, 1488 differetially expressed lncRNAs and 1460 differentially expressed mRNAs were identified using the Affymetrix Human oelncRNA Array. A co-expression network of the identified lncRNAs and mRNAs was constructed. In this network, lncRNA lnc-RP11-1220 K2.2.1–7 is correlated with mRNA CXCR1 and CLEC4D; lncRNA lnc-ANXA3–2 is correlated with mRNA CLEC4D; lncRNA lnc-TRAPPC5–1 is correlated with mRNA DYSF and HLX; lncRNA lnc-ZNF638–1 is correlated with mRNA DYSF and HLX. Significantly different expressions between pediatric sepsis patients and controls were validated by qPCR for the 4 lncRNAs (lncRNA lnc-RP11-1220 K2.2.1–7, lncRNA lnc-ANXA3–2, lncRNA lnc-TRAPPC5–1, lncRNA lnc-ZNF638–1) and 4 co-expressed mRNAs (CXCR1, CLEC4D, DYSF, HLX), validating the microarray results. CXCR1 is one of the major chemokine receptors on polymorphionuclear neutrophils [23], and the involvement of polymorphionuclear neutrophils in sepsis is well recognized [24–27].CLEC4D, a member of Dectin-2 family, functions in resolution of inflammation, possibly through facilitating neutrophil turnover [28]. DYSF, a type II transmembrane protein, is involved in muscle membrane repair [29, 30], and also functions in the regulation of cellular adhesion in human monocytes [31]. HLX is known to be a marker of immature hematopoietic cells, and it also plays a role in the activation of T lymphocyte or natural killer cells [32, 33]. HLX is reported to be involved in the macrophage differentiation process as well [34]. Enrichment analysis of the co-expression network suggested that mRNAs in the co-expression network were enriched in innate immune response. These results indicated that lncRNAs of the co-expression network correlated with mRNA expression and involved in the pathogenesis of sepsis. Compared with prior study - FEBS Open Bio. 2019; 9(1): 148–158, our analysis identified novel lncRNA – mRNA pairs that play roles in sepsis.

In addition, it is important to note that this study has several limitations. First, the small sample size required more careful consideration on our analysis. Second, considering the influence of gender and age on pediatric sepsis patients has been reported [35], subgroup analyses based on potential influential factors (such as age, gender, and disease severity) are needed in future research. Third, little is known about the molecular functions of these candidate lncRNAs in the development of pediatric sepsis, more in vivo / in vitro research is therefore needed to be carried out in the future.

## Conclusions

Here, we reported a total of 1488 differetially expressed lncRNAs and 1460 differentially expressed mRNAs in pediatric patients with sepsis. A co-expression network of these lncRNAs and mRNAs was constructed and suggested that lncRNAs of the co-expression network correlated with mRNA expression and involved in the pathogenesis of sepsis. Our study contributes to a comprehensive understading of the involvment of lncRNAs and mRNAs in pediatric sepsis, which may guide subsequent experimental research. Furthermore, our study may also provide potential candidate lncRNAs and mRNAs for the diagnosis and treatment of pediatric sepsis.

## Supplementary information

**Additional file 1.** Clinical parameters of the sepsis and control groups used in both microarray and qPCR validation.

**Additional file 2.** Primers designed for validation of lncRNA & mRNA expression patterns by qPCR.

**Additional file 3.** 1488 differentially expressed lncRNAs (996 upregulated; 492 downregulated) identified using the Affymetrix Human oelncRNA Array between pediatric sepsis group and control group.

**Additional file 4.** 1460 differentially expressed mRNAs (1018 upregulated; 442 downregulated) identified using the Affymetrix Human oelncRNA Array between pediatric sepsis group and control group.

**Additional file 5.** Top performing mRNAs in diagnostic prediction of pediatric sepsis.

**Additional file 6.** Conservation analysis results of the 4 lncRNAs.

## Data Availability

The datasets used and/or analysed during the current study are available in Gene Expression Omnibus (GEO) database [GSE145227].

## Abbreviations

AUC: Area under the curve
CLEC4D: C-type lectin domain family 4 member D
CXCR1: C-X-C motif chemokine receptor 1
DYSF: Dysferlin
FDR: False discovery rate
GO: Gene ontology
HLX: H2.0 like homeobox
KEGG: Kyoto Encyclopedia of Genes and Genomes
LFC: Log2 Fold Change
LncRNAs: Long noncoding RNAs
MNCs: Mononuclear cells
QPCR: Quantitative real-time PCR
RMA: Robust Multichip Average
ROC: Receiver operating characteristic

## References

[1] Lee ST, Chu K, Im WS, Yoon HJ, Im JY, Park JE, Park KH, Jung KH, Lee SK, Kim M, Roh JK (2011). Altered microRNA regulation in Huntington's disease models. Exp Neurol.

[2] Wang KC, Chang HY (2011). Molecular mechanisms of long noncoding RNAs. Mol Cell.

[3] Rinn JL, Chang HY (2012). Genome regulation by long noncoding RNAs. Annu Rev Biochem.

[4] Pan B, Shi ZJ, Yan JY, Li JH, Feng SQ (2017). Long non-coding RNA NONMMUG014387 promotes Schwann cell proliferation after peripheral nerve injury. Neural Regen Res.

[5] Marques-Rocha JL, Samblas M, Milagro FI, Bressan J, Martínez JA, Marti A (2015). Noncoding RNAs, cytokines, and inflammation-related diseases. FASEB J.

[6] Ren Gui‐Ling, Zhu Jie, Li Jun, Meng Xiao‐Ming (2018). Noncoding RNAs in acute kidney injury. Journal of Cellular Physiology.

[7] Li L, Wang L, Li H, Han X, Chen S, Yang B, Hu Z, Zhu H, Cai C, Chen J, Li X, Huang J, Gu D (2018). Characterization of LncRNA expression profile and identification of novel LncRNA biomarkers to diagnose coronary artery disease. Atherosclerosis.

[8] Ho J, Chan H, Wong SH, Wang MH, Yu J, Xiao Z, Liu X, Choi G, Leung CC, Wong WT, Li Z, Gin T, Chan MT, Wu WK (2016). The involvement of regulatory non-coding RNAs in sepsis: a systematic review. Crit Care.

[9] Huang S, Qian K, Zhu Y, Huang Z, Luo Q, Qing C (2017). Diagnostic value of the lncRNA NEAT1 in peripheral blood mononuclear cells of patients with Sepsis. Dis Markers.

[10] Kissoon N, Carapetis J (2015). Pediatric sepsis in the developing world. J Inf Secur.

[11] Gautier L, Cope L, Bolstad BM, Irizarry RA (2004). Affy--analysis of Affymetrix GeneChip data at the probe level. Bioinformatics.

[12] Smyth GK. limma: Linear Models for Microarray Data. In: Gentleman R, Carey V, Huber W, Irizarry R, Dudoit S. Bioinformatics and Computational Biology Solutions Using R and Bioconductor. New York: Springer; 2005. p. 397–420.

[13] Ritchie ME, Phipson B, Wu D, Hu Y, Law CW, Shi W (2015). limma powers differential expression analyses for RNA-sequencing and microarray studies. Nucleic Acids Res.

[14] Huang da W, Sherman BT, Lempicki RA (2009). Systematic and integrative analysis of large gene lists using DAVID bioinformatics resources. Nat Protoc.

[15] Huang da W, Sherman BT, Lempicki RA (2009). Bioinformatics enrichment tools: paths toward the comprehensive functional analysis of large gene lists. Nucleic Acids Res.

[16] Falcon S, Gentleman R (2007). Using GOstats to test gene lists for GO term association. Bioinformatics.

[17] Kanehisa M, Goto S (2000). KEGG: Kyoto encyclopedia of genes and genomes. Nucleic Acids Res.

[18] Robin X, Turck N, Hainard A, Tiberti N, Lisacek F, Sanchez JC (2011). pROC: an open-source package for R and S+ to analyze and compare ROC curves. BMC Bioinformatics.

[19] Shannon P, Markiel A, Ozier O, Baliga NS, Wang JT, Ramage D (2003). Cytoscape: a software environment for integrated models of biomolecular interaction networks. Genome Res.

[20] Siepel A, Bejerano G, Pedersen JS, Hinrichs AS, Hou M, Rosenbloom K (2005). Evolutionarily conserved elements in vertebrate, insect, worm, and yeast genomes. Genome Res.

[21] Chatterjee P, Roy D, Bhattacharyya M, Bandyopadhyay S (2017). Biological networks in Parkinson's disease: an insight into the epigenetic mechanisms associated with this disease. BMC Genomics.

[22] Chatterjee P, Bhattacharyya M, Bandyopadhyay S, Roy D (2014). Studying the system-level involvement of microRNAs in Parkinson's disease. PLoS One.

[23] Takahashi M, Ishiko T, Kamohara H, Hidaka H, Ikeda O, Ogawa M, Baba H (2007). Curcumin (1,7-bis (4-hydroxy-3-methoxyphenyl)-1,6-heptadiene-3,5-dione) blocks the chemotaxis of neutrophils by inhibiting signal transduction through IL-8 receptors. Mediat Inflamm.

[24] Drifte G, Dunn-Siegrist I, Tissières P, Pugin J (2013). Innate immune functions of immature neutrophils in patients with sepsis and severe systemic inflammatory response syndrome. Crit Care Med.

[25] Boppana NB, Devarajan A, Gopal K, Barathan M, Bakar SA, Shankar EM, Ebrahim AS, Farooq SM (2014). Blockade of CXCR2 signalling: a potential therapeutic target for preventing neutrophil-mediated inflammatory diseases. Exp Biol Med (Maywood).

[26] Jaillon S, Galdiero MR, Del Prete D, Cassatella MA, Garlanda C, Mantovani A (2013). Neutrophils in innate and adaptive immunity. Semin Immunopathol.

[27] Malkin AD, Sheehan RP, Mathew S, Federspiel WJ, Redl H, Clermont G (2015). A neutrophil phenotype model for extracorporeal treatment of Sepsis. PLoS Comput Biol.

[28] Steichen AL, Binstock BJ, Mishra BB, Sharma J (2013). C-type lectin receptor Clec4d plays a protective role in resolution of gram-negative pneumonia. J Leukoc Biol.

[29] Bansal D, Miyake K, Vogel SS, Groh S, Chen CC, Williamson R, McNeil PL, Campbell KP (2003). Defective membrane repair in dysferlin-deficient muscular dystrophy. Nature.

[30] Lennon NJ, Kho A, Bacskai BJ, Perlmutter SL, Hyman BT, Brown RH (2003). Dysferlin interacts with annexins A1 and A2 and mediates sarcolemmal wound-healing. J Biol Chem.

[31] de Morrée A, Flix B, Bagaric I, Wang J, van den Boogaard M, Grand Moursel L, Frants RR, Illa I, Gallardo E, Toes R, van der Maarel SM (2013). Dysferlin regulates cell adhesion in human monocytes. J Biol Chem.

[32] Deguchi Y, Kirschenbaum A, Kehrl JH (1992). A diverged homeobox gene is involved in the proliferation and lineage commitment of human hematopoietic progenitors and highly expressed in acute myelogenous leukemia. Blood..

[33] Becknell B, Hughes TL, Freud AG, Blaser BW, Yu J, Trotta R, Mao HC (2007). Caligiuri de Jesús ML, Alghothani M, Benson DM Jr, Lehman a, Jarjoura D, Perrotti D, bates MD, Caligiuri MA. Hlx homeobox transcription factor negatively regulates interferon-gamma production in monokine-activated natural killer cells. Blood.

[34] Baek YS, Haas S, Hackstein H, Bein G, Hernandez-Santana M, Lehrach H, Sauer S, Seitz H (2009). Identification of novel transcriptional regulators involved in macrophage differentiation and activation in U937 cells. BMC Immunol.

[35] Maat M, Buysse CM, Emonts M, Spanjaard L, Joosten KF, de Groot R (2007). Improved survival of children with sepsis and purpura: effects of age, gender, and era. Crit Care.

