# Supplementary material for: Long noncoding RNA and messenger RNA abnormalities in pediatric sepsis: a preliminary study
Source: BMC Med Genomics. 2020 Mar 10;13:36. doi: 10.1186/s12920-020-0698-x (PMC7063742; doi:10.1186/s12920-020-0698-x)
Supplement: Supplementary file 1 — Additional file 1. Clinical parameters of the sepsis and control groups used in both microarray and qPCR validation. [file 12920_2020_698_MOESM1_ESM.pdf]

Clinical parameters	Control	Sepsis	*P*
Hemoglobin, g/L	120 [116.3 - 128.5]	92.5 [79.3 - 105]	< 0.001
Platelet, 10^9/L #	293.8 ± 22.8	196.2 ± 33.9	0.038
Albumin, g/L #	44.1 ± 0.6	35.1 ± 2.7	0.002
Blood urea, mmol/L	3.9 [3.5 - 4.1]	3.2 [2.4 - 13.6]	0.759
Creatinine, μmol/L	23 [20 - 24.7]	27.9 [22.5 - 72.7]	0.136
CRP, mg/L	0.2 [0.1 - 0.5]	104.9 [3.7 - 254.8]	< 0.001
LDH, U/L	332.8 [272.5 - 368.6]	530.5 [419.7 - 831.9]	< 0.001
WBC, 10^9/L	8.6 [6.3 - 12]	5.2 [3.5 - 13.6]	0.314
Neutrophil, 10^9/L	2 [1.3 - 3]	2.9 [1.9 - 9.5]	0.159

Data are presented as the mean ± standard error of mean.

CRP, C-reactive protein; LDH, lactate dehydrogenase; WBC, white blood cell.

Continuous variables were compared using Student's t-test or Mann-Whitney's *U* test, depending on the distribution, and were presented as the mean ± standard error of mean or median (interquartile range).
