# Supplementary material for: Long noncoding RNA and messenger RNA abnormalities in pediatric sepsis: a preliminary study
Source: BMC Med Genomics. 2020 Mar 10;13:36. doi: 10.1186/s12920-020-0698-x (PMC7063742; doi:10.1186/s12920-020-0698-x)
Supplement: Supplementary file 2 — Additional file 2. Primers designed for validation of lncRNA & mRNA expression patterns by qPCR. [file 12920_2020_698_MOESM2_ESM.pdf]

Gene name	Forward and reverse primer	Tm (℃)	Product length (bp)
β-actin	F:5'CATGTACGTTGCTATCCAGGC3'<br>R:5'CTCCTTAATGTCACGCACGAT3'	60	249
HLX	F:5'ATCCCATTAACGAGGCTTCTG3'<br>R:5'CAGCATAGGGACCTGGAAAC3'	60	102
DYSF	F:5'CTGGAGATTGGTTCGTGTGT3'<br>R:5'TCTGGTTCTCAAACACCTCTTC3'	60	93
CXCR1	F:5'CGCCATGGATTCCTCAAGAT3'<br>R:5'AGAGACATTGACAGACGAAGAAG3'	60	105
CLEC4D	F:5'CCAGCTGATACCTTCGGTTATT3'<br>R:5'ACTCCTGTGCCTCTCTTACA3'	60	117
lnc-ZNF638-1	F:5'TGCTCTGGGTGAAGTGTC3'<br>R:5'AGCCTCTATTCCTTGAGGGTA3'	60	76
lnc-TRAPPC5-1	F:5'AGAGACAGTGAGGATGGTT3'<br>R:5'AAGAGGCAGCAAAGTGAC3'	60	62
lnc-ANXA3-2	F:5'CAGCCTTTAATGATGCCGT3'<br>R:5'TGTACTCATGGAGCGCAC3'	60	74
lnc-RP11-1220K2.2.1-7	F:5'AAACATCCATCTACATGCTGAG3'<br>R:5'CTTTACCCAGGTTCTGCTATGA3'	60	71
