# Supplementary figures and images for: Long noncoding RNA and messenger RNA abnormalities in pediatric sepsis: a preliminary study
Source: BMC Med Genomics. 2020 Mar 10;13:36. doi: 10.1186/s12920-020-0698-x (PMC7063742; doi:10.1186/s12920-020-0698-x)

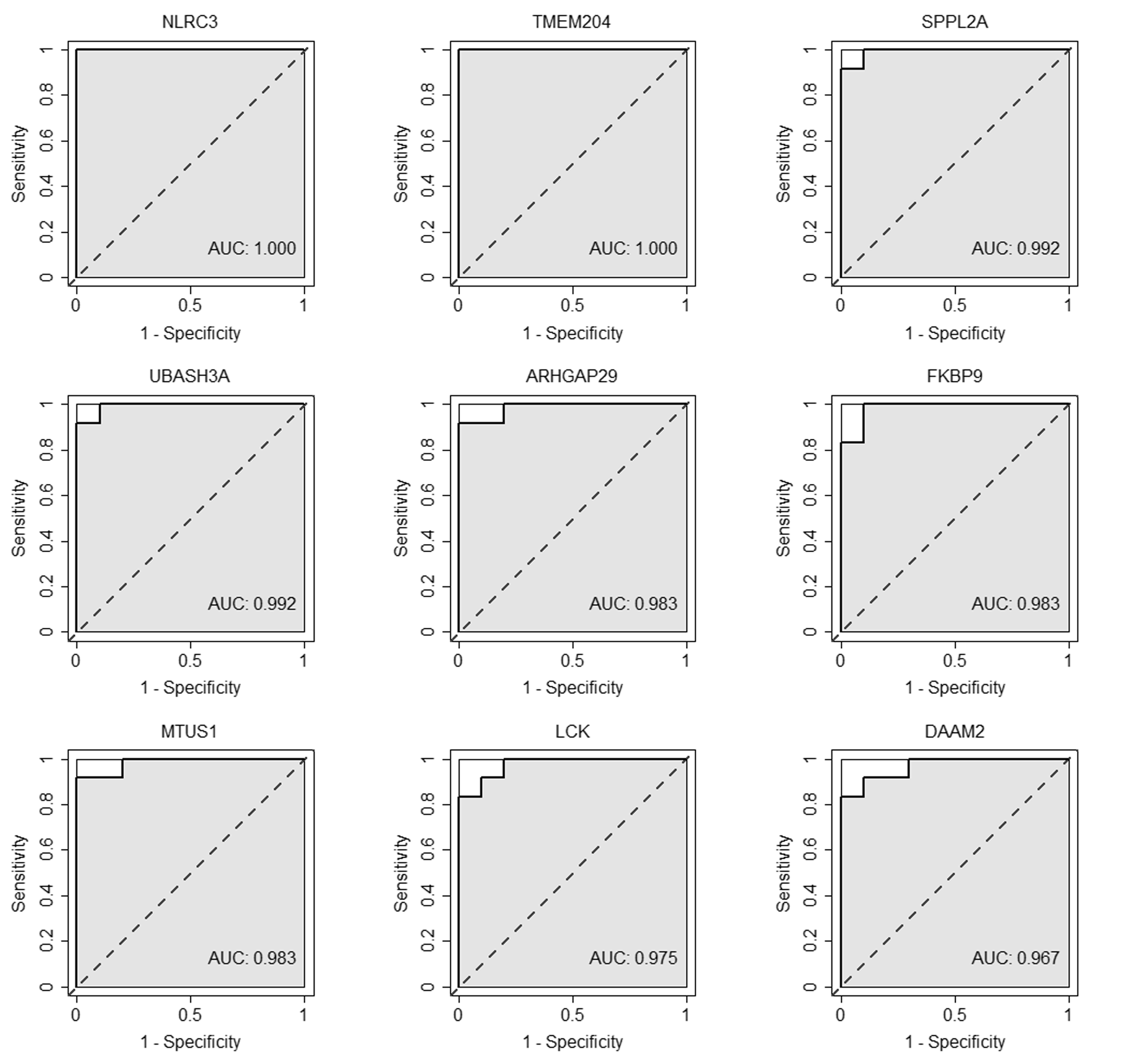

Supplement: Supplementary file 5 — Additional file 5. Top performing mRNAs in diagnostic prediction of pediatric sepsis. [file 12920_2020_698_MOESM5_ESM.png]
